# Unveiling the Therapeutic Potential of Squalene Synthase: Deciphering Its Biochemical Mechanism, Disease Implications, and Intriguing Ties to Ferroptosis

**DOI:** 10.3390/cancers15143731

**Published:** 2023-07-22

**Authors:** David Figueredo Picón, Rachid Skouta

**Affiliations:** Department of Biology, University of Massachusetts, Amherst, MA 01003, USA; dfigueredopi@umass.edu

**Keywords:** squalene synthase (SQS), cholesterol, ferroptosis, lipid peroxidation, small-molecule modulators of SQS

## Abstract

**Simple Summary:**

Squalene synthase is a key enzyme that not only participates in the mevalonate pathway, resulting in cholesterol, but also acts as a potential ferroptosis regulator. The synthesis of squalene and its release in the endoplasmic reticulum protects the cell against lipid peroxidation, resulting in cell survival. We aimed to investigate the therapeutic potential of this enzyme based on its biochemical structure, reaction mechanism, significance in diseases, and relation to ferroptosis. In addition, we compiled the known squalene synthase inhibitors, aiming to provide useful information for researchers and to encourage the conduction of more studies on the therapeutics of squalene synthase.

**Abstract:**

Squalene synthase (SQS) has emerged as a promising therapeutic target for various diseases, including cancers, owing to its pivotal role in the mevalonate pathway and the antioxidant properties of squalene. Primarily, SQS orchestrates the head-to-head condensation reaction, catalyzing the fusion of two farnesyl pyrophosphate molecules, leading to the formation of squalene, which has been depicted as a highly effective oxygen-scavenging agent in in vitro studies. Recent studies have depicted this isoprenoid as a protective layer against ferroptosis due to its potential regulation of lipid peroxidation, as well as its protection against oxidative damage. Therefore, beyond its fundamental function, recent investigations have unveiled additional roles for SQS as a regulator of lipid peroxidation and programmed cell death pathways, such as ferroptosis—a type of cell death characterized by elevated levels of lipid peroxide, one of the forms of reactive oxygen species (ROS), and intracellular iron concentration. Notably, thorough explorations have shed light on the distinctive features that set SQS apart from other members within the isoprenoid synthase superfamily. Its unique biochemical structure, intricately intertwined with its reaction mechanism, has garnered significant attention. Moreover, considerable evidence substantiates the significance of SQS in various disease contexts, and its intriguing association with ferroptosis and lipid peroxidation. The objective of this report is to analyze the existing literature comprehensively, corroborating these findings, and provide an up-to-date perspective on the current understanding of SQS as a prospective therapeutic target, as well as its intricate relationship with ferroptosis. This review aims to consolidate the knowledge surrounding SQS, thereby contributing to the broader comprehension of its potential implications in disease management and therapeutic interventions.

## 1. Introduction

The mevalonate pathway is essential for the biosynthesis of all mammalian isoprenoids. It produces crucial metabolites for the post-translational addition of hydrophobic molecules (prenylation) to guanosine-triphosphate-binding proteins [1]. Moreover, these metabolites are fundamental for many cellular processes, including cholesterol synthesis [1,2]. Cholesterol has been closely related to ferroptosis, an intracellular iron-dependent form of cell death. Cholesterol metabolites’ availability has shown to increase the capacity of tumor and metastatic cells by upregulating cellular uptake and lipid biosynthesis. This correlation is due to cholesterol’s key role in cell membrane formation, as well as its susceptibility to oxidants, such as hydroxyl radicals. One of the intermediates in the cholesterol biosynthesis pathway, squalene, is considered a possible regulator of lipid peroxidation by the modification of the cellular lipid profile. Additionally, squalene lessens oxidative damage by reducing the levels of reactive oxygen species. These two squalene functions ultimately regulate programmed cell death, such as ferroptosis. Therefore, this review will focus primarily on one of the downstream enzymes in the mevalonate pathway, squalene synthase (SQS). This member of the isoprenoid synthase superfamily catalyzes the formation of a lipophilic cholesterol intermediate, squalene, which has been associated with many health disorders, so researchers have investigated SQS as a potential therapeutic target.

## 2. Squalene Synthase (SQS)

### 2.1. The Origins of SQS

**Scheme 1:**
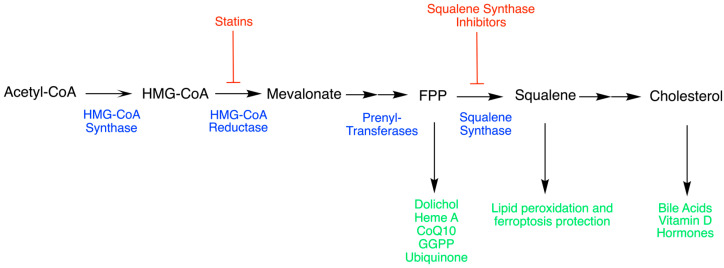
Mevalonate pathway. Intermediates are shown in black, enzymes in blue, inhibitors in red, and biological usage in green.

Phylogenetic studies on SQS, also known as farnesyl-diphosphate farnesyl transferase, have shown its relevancy in evolution, as it originated in bacteria and has now been identified in all living organisms [2,3]. This enzyme is a key component of the isoprenoid metabolic pathway, leading to the synthesis of eukaryotic sterols and bacterial hopanoids [4]. Its mRNA expression has been identified in most human organs; however, expression levels vary between them, with the highest being in the testis and skeletal muscle, while levels are low in the liver [5]. Within the cell, SQS is exclusively located in the smooth endoplasmic reticulum membrane, as observed in normal rat livers with immunoelectron microscopy [6]. Additionally, it is associated with the final branch point of the cholesterol biosynthesis pathway, also known as the mevalonate pathway, shown in Scheme 1. The reaction catalyzed by SQS is divided into two half-reactions: the first half-reaction results in the formation of cyclopropylcarbonyl pyrophosphate (presqualene pyrophosphate or PSPP) from two identical soluble allylic molecules of farnesyl pyrophosphate (FPP, C_15_). This intermediate, PSPP, mediates the inflammatory response in neutrophils, where it inhibits oxygen radical production [7]. In the second half-reaction, PSPP is rearranged into squalene, an insoluble isoprenoid compound with a C_30_H_50_ molecular formula that serves as the precursor for hormones, bile acids, and vitamin D, as shown in Scheme 1 [6]. In addition to the mevalonate pathway, squalene is part of the sterol biosynthesis pathway. Therefore, squalene can take two possible routes after forming squalene epoxide via squalene epoxidase (SQLE): follow the mevalonate pathway to form cholesterol or follow the squalene dioxide pathway to form oxysterols [6,8].

Squalene was originally found in shark liver oil in 1926 by the Scottish chemist Isidor Morriss Heibron and his colleagues, even though Morris reported that Mitsumaru Tsujimoto was the first to isolate squalene in 1916 [9]. The substrate stereochemistry of SQS was established in 1966 by George Popják and colleagues when studying the mechanism from farnesyl pyphosphate and presqualene pyrophosphate to squalene [10]. In 1976, substrate analogs helped researchers differentiate between the two substrate-binding sites within the enzyme [11]. In 1980, researchers discovered the substrate specificity of SQS, noting that the 3-methyl of farnesyl pyrophosphate and the 10,11-double bond are necessary for proper activity [12]. However, it was not until 1988 that Sasiak and Rilling realized that two reactions in the pathway were catalyzed by a single enzyme, SQS, while purifying SQS to homogeneity from *Saccharomyces cerevisiae* [13]. Later on, in 1992 and 1993, rat SQS and human SQS were both cloned through cDNA using the same group of primers for both [14,15]. Lastly, in 2000, the crystal structure of human SQS was determined for the first time by Pandit et al. [8]. Since then, researchers have recognized the therapeutic potential of SQS and have worked on discovering regulators.

### 2.2. Isoprenoid Synthase Superfamily

SQS is a member of a superfamily of phylogenetically related enzymes involved in synthesizing isoprenoids or terpenoids, considered the largest class of natural products on earth, present in all living organisms [16]. Within the isoprenoid synthase superfamily, SQS belongs to the isoprenoid synthase type I family, which is characterized by carbocation formation through the release of a phosphate group [17]. SQS shares some similarities with other class I isoprenoid biosynthetic enzymes such as farnesyl pyrophosphate synthase (FPS), which catalyzes the synthesis of 2-trans,6-trans-farnesyl pyrophosphate, SQS substrate [9]. The frequent presence of DDXXD aspartate-rich sequence motif regions (Asp-RR) allows the pyrophosphate groups to bind through Mg2^+^ substrates, leading to the releasing of a phosphate group [6]. SQS is structurally homologous compared with other isoprenoid biosynthetic enzymes, as they share an α-helical core surrounding an inner channel and contain highly conserved aspartate-rich regions [6]. Overall, these similarities across the isoprenoid synthase superfamily, specifically type I, suggest evolution with similar structures through divergence from a common predecessor, regardless of the differences in the amino acid sequence.

### 2.3. General Mechanism of SQS

**Scheme 2:**
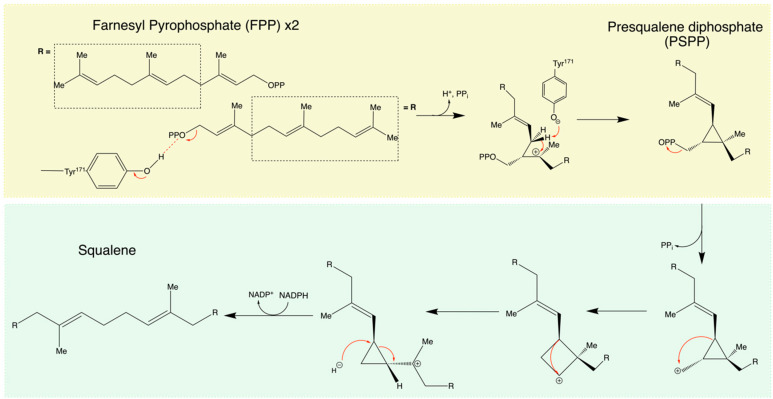
SQS reaction mechanism. The top part represents the first half-reaction carried out by SQS in which a molecule of PSPP is formed from the condensation of two FPP molecules. The bottom part represents the second half-reaction in which PSPP is reduced and reorganized, which results in squalene.

The reaction catalyzed by SQS occurs during the final stage of the isoprenoid biosynthesis pathway and is considered the first committed step in the mevalonate pathway, as shown in Scheme 1. Even though it is known that the SQS mechanism occurs in two distinct half-reactions, the exact mechanism of this enzyme is still being discussed, but one model proposed by the investigators Poulter and Rilling is presented here [14]. SQS catalyzes squalene biosynthesis, a terpene synthesis, by the reductive dimerization of two FPP molecules [14]. The biochemical structure of human SQS, see Section 2.4, reveals that there are two hydrophilic active sites and an inhibitory pocket where the second half-reaction takes place. This difference in environment proposes a mechanism in which FPP molecules bind at the hydrophilic end of the channel. Once the intermediate, presqualene pyrophosphate (PSQPP), is formed, it is then moved to the hydrophobic pocket [6]. This implies that the catalytic site for the first half-reaction is in the hydrophilic end of the channel and for the second half-reaction in the hydrophobic end [6]. SQS’s catalyzation is unique due to both consecutive steps, including forming an allylic carbocation intermediate [6]. The overall reaction is carried out through a tail-to-tail terpene synthesis, which is a condensation of two farnesyl pyrophosphate units joined at the carbon 4 (C_4_) and forming a 4-4’ linkage, respectively. This reaction progresses from two C_15_ reagents to a C_30_ isoprenoid product, squalene [17]. For this head-to-head orientation to occur, atypical to other prenyltransferases, a divalent cation (Mg^2+^) is required to stabilize the two phosphate groups in FPP at the hydrophilic end [6]. In the first half-reaction, two molecules of FPP react, forming more stable intermediate, cyclopropylcarbonyl pyrophosphate (PSQPP), also referred as presqualene pyrophosphate [18]. It has been speculated that one of the FPP molecules acts as a donor by ionizing and forming an allylic carbocation that is then attacked by the nucleophilic 2–3 bond of the acceptor FPP molecule [6]. During the formation of PSQPP, a proton and a molecule of inorganic phosphate (PPi) are released. Once the PSQPP intermediate is formed, it is then moved to the hydrophobic pocket through the inner channel without dissociation from SQS [6]. During the second half-reaction, thought to occur in the hydrophobic pocket of the inner channel, cleavage of two cyclopropane bonds and, therefore, ionization of the diphosphate group in PSQPP takes place to give a cyclopropylcarbonyl cation [19,20]. Once the cation is formed, it follows a bond migration rearrangement between C1 and C2, resulting in a cyclobutyl carbocation. Thereafter, a second 1,2-migration takes place to form a cyclopropylcarbonyl cation, in this case, a tertiary carbocation intermediate. Lastly, the formed tertiary carbocation is reduced by nicotinamide adenine dinucleotide phosphate (NADPH), causing the opening of the ring, resulting in squalene, which is then released into the membrane of the endoplasmatic reticulum [21]. It is also noted that, during the second half-reaction, PPi, NADP+, and H+ are released, as shown in Scheme 2.

### 2.4. The Biochemical Structure of Human SQS

**Figure 1:**
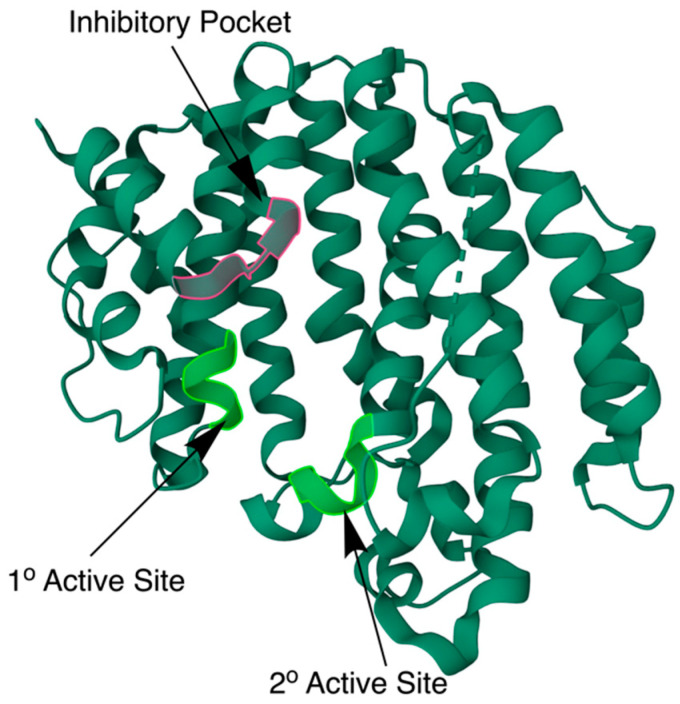
Crystal structure of the human SQS protein adapted from the Protein Data Bank (PDB identification code: 3ASX). The structure was originally reported by (https://pubmed.ncbi.nlm.nih.gov/21353782/, accessed on 15 February 2023). Shows the full crystal structure of SQS, including the two aspartate-rich regions (Asp-RR) (^80^DTLED^84^ and ^219^DYLED^223^) acting as active sites in green, and the residues 50 through 54 forming the hydrophobic pocket or inhibitor-binding pocket in red.

The farnesyl-diphosphate farnesyltransferase 1 (FDFT1) gene, located in chromosome 8, 8p22-p23.1 region, encodes human squalene synthase (hSQS) [22]. This gene contains 10 exons, which lead to 11 different isoforms that encode five different proteins, with their main differences being in the N-terminus [23]. Its open reading frame has 1248 nucleotides, which encode 415 amino acids, resulting in a molecular weight of 47.16 kDa [23]. Its isoelectric point is 6.50, the pH at which the net isoelectric charge of SQS becomes neutral due to the presence of 56 negatively charged and 43 positively charged amino acids [23]. The structure of this enzyme is composed of one mostly alpha-helical domain, as shown in Figure 1. Regarding its half-life, previous studies state that it is about 30 h [5].

**Figure 2:**
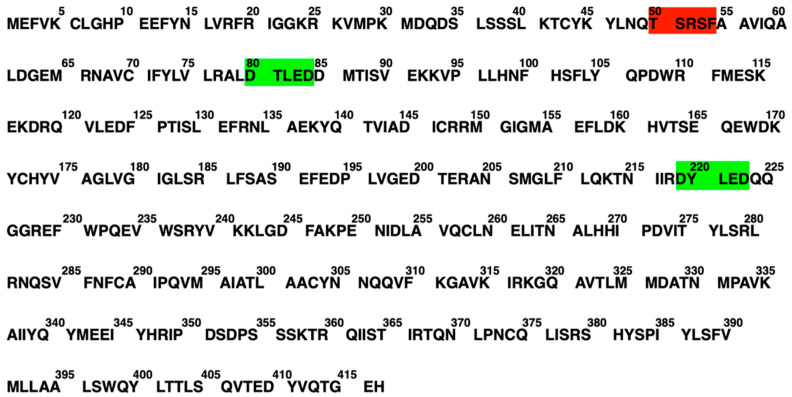
Amino acid sequence for SQS, imported from http://www.uniprot.org/uniprot/P37268, accessed on 29 April 2023 using the UniProt-XML importer. The figure is color-coded by the two aspartate-rich regions (Asp-RR), ^80^DTLED^84^ and ^219^DYLED^223^, and the inhibitor-binding pocket or hydrophobic pocket formed by residues 50 through 54 in red and green, respectively.

The crystal structure in Figure 1 shows how hSQS is divided into different layers, each containing different alpha helices connected by loops [24]. The enzyme is embedded in the membrane of the endoplasmic reticulum at the C-terminus, where a membrane-spanning domain is found [25]. Meanwhile, the N-terminus protrudes into the cytoplasm, where the two soluble FPP molecules bind. Additionally, the monomeric protein encloses an inner channel where the reductive dimerization of two 2-trans,6-trans-farnesyl pyrophosphate (FPP) molecules is catalyzed [6]. The intracellular location of SQS, including its catalytic domain (N-terminus) projecting into the cytosol, was determined after the ability to release an active form of SQS from microsomal membranes by trypsin lysis was discovered [6]. This orientation of the enzyme allows access to the water-soluble FPP molecules and NADPH from the cytosol, and the release of squalene into a hydrophobic environment [6]. On the lower end of the inner channel, protruding the cytoplasm, two aspartate-rich regions (Asp-RR) (^80^DTLED^84^ and ^219^DYLED^223^), see Figure 2, are located on opposite walls of the channel, similarly but not exactly following the DDXXD consensus sequence across the isoprenoid synthase superfamily [26]. These residues are one of the most highly conserved regions of the amino acid sequence, as the two Asp-RR on SQS overlap with those on farnesyl pyrophosphate synthase (FPS), Asp^117^–Asp^121^ and Asp^257^–Asp^261^. Based on the structural superposition of SQS with other enzymes in the same family, it has been suggested that the two Asp-RR sequentially bind the pyrophosphates of the two FPP molecules via Mg^2+^ binding during the first half-reaction. Meanwhile, the isoprene tails of both FPP molecules stick up towards the hydrophobic end of the inner channel. This theory is supported by the complete loss of function when a mutation on Asp^219^ and Asp^223^ occurs or by how a similar result is predicted when affecting the ^80^DTLED^84^ region based on the crystal structure, as shown in Figure 1. Once bound to the two Asp-RR, during the first half-reaction, one FPP is ionized by removing the pyrophosphate group to form an allylic carbocation. This is possible due to the proton served by a conserved tyrosine residue (Tyr^171^), which becomes a phenolate anion after donating the proton. Once ionized, the acceptor FPP molecule attacks the allylic carbocation to form a tertiary carbocation, which becomes PSQPP by donating a proton to the previously formed phenolate anion. The product of the first half-reaction at the lower end of the inner channel is PSQPP, and it is obtained through the usage of two Asp-RR and a tyrosine residue (Tyr^171^), which are conserved sequences. The crystal structure of hSQS also explains how a large hydrophobic cavity is located on the same side of the inner channel as the ^80^DTLED^84^ motif, taking in the 30-carbon intermediate (PSQPP). This motif suggests that the prenyl acceptor is in the lower and hydrophilic end of the inner channel. This form was confirmed by the X-ray structures obtained from FPS in which the growing isoprenyl was bound to Asp^117^–Asp^121^, corresponding to ^80^DTLED^84^ in SQS. The conserved arginine amino acids Arg^218^ and Arg^228^ adjacent to the second Asp-RR, 219DYLED223, will likely stabilize the pyrophosphate leaving when the two FPP molecules are condensed during the first half-reaction.

**Figure 3:**
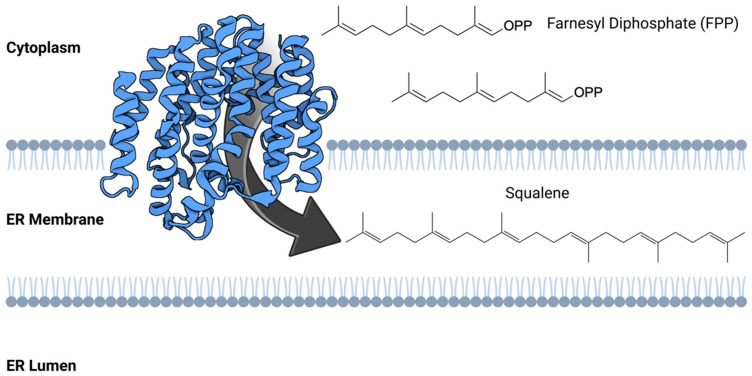
Localization of SQS, reactants, and products. Farnesyl pyrophosphate (FPP) is located on the cytoplasm. Two molecules independently bind to the hydrophilic end of the inner channel where the first half-reaction takes place. The intermediate, presqualene pyrophosphate (PSPP), is moved through the inner channel towards the hydrophobic end, releasing squalene into the endoplasmatic reticulum (ER) membrane.

The top end of SQS, completely embedded in the ER membrane, is the only part of the inner channel that is not exposed to solvent, and it is covered by a flap made up of residues 50 through 54, shown in Figure 3 [9]. Moreover, the side chain of Phe^54^ forms one wall of a large hydrophobic cavity under the flap, an inhibitor-binding pocket in which inhibitors bind [8]. Inhibition of SQS has been previously achieved by filling up the inhibitor-binding pocket with a bulky hydrophobic group [9]. This hydrophobic pocket also acts as the opening to the solvent-exposed part of the inner channel [9]. Once PSQPP is produced at the lower end of the enzyme, it is moved higher in the inner channel to prevent its interaction with water, as it is a reactive intermediate [9]. To obtain squalene from PSQPP, several carbocation rearrangements need to take place inside the hydrophobic pocket so that squalene can then be released into the membrane of the endoplasmic reticulum, as shown in Figure 3.

The location at which the second half-reaction occurs is not yet clear, but several ideas based on known mechanisms have been proposed. Since a reactive carbocation intermediate also participates during the second half-reaction, hSQS uses its hydrophobic pocket in the inner channel to accommodate these carbocationic intermediates [9]. The residues conforming to this pocket are predominately hydrophobic as they prevent the carbocations from being exposed to the solvent prematurely [9]. This pocket is also a conserved sequence across all known SQS amino acid sequences. Mutations in some of these specific residues, for example, Phe^288^, show a complete loss of activity in the second reaction since it is likely to stabilize one of the carbocationic intermediates of the second half-reaction [9]. Even though there is no evidence of a motif associated with nucleotide binding in the structure, the ^314^VKIRK^318^ domain has been suggested as the NADPH binding site for the second half-reaction. This domain is conserved in SQS across different species; however, it is not conserved in other enzymes in the class I isoprenoid biosynthetic family, which do not require binding a nucleotide co-factor [17]. Overall, it can be concluded that the two catalytic sites are different in the environment. The active site participating in the first half-reaction, and therefore where the two FPP molecules initially bind, is at the hydrophilic lower end of the inner channel closer to the cytoplasm. In contrast, the active site participating in the second half-reaction is hydrophobic and located within the ER membrane. These differences in environment explain how SQS is embedded in the membrane of the ER and how the lipophilic product, squalene, leaves the enzyme through the upper end closer to the membrane.

### 2.5. Mutations

#### 2.5.1. Natural Mutations of SQS

Since the discovery of SQS by George Popják in 1966, not many natural mutations have been identified besides a rare inborn error in the FDFT1 gene, resulting in squalene synthase deficiency (SQSD). This mutation results in a rare autosomal recessive disorder so far identified in three European newborns. [24,27]. This disorder presents with multi-system phenotypes, including facial dysmorphism, generalized seizure disorder, structural brain malformations, and profound intellectual disability [28]. The molecular genetics of SQSD involves two out of the three diagnosed individuals presenting with a 120 kb deletion on the maternal chromosome 8, which includes exons 6 through 10 of the FDFT1 gene, along with a TC deletion and AG insertion within the FDFT1 gene in the paternal chromosome [28]. Similarly, the third SQSD patient was found to be carrying another TC deletion and AG insertion in the FDFT1 gene [28]. All three patients share a splicing defect in chromosome 8 [24]. Western blot studies in SQSD patients have shown a significant reduction in the FDFT1 protein compared with a healthy individual [24]. SQSD has been shown to be more detectable using sequence analysis to visualize the genetic mutation [28].

#### 2.5.2. Artificial Mutations of SQS

The central cavity of hSQS, where the two conserved Asp-RR are found, has large amino acids adjacent, such as Val^69^, Leu^183^, Phe^187^, Tyr^276^, and Phe^288^ [28]. Previous studies have shown that substituting Phe288 with charged residues such as alanine downregulates the enzymatic activity of SQS, suggesting that Phe288 is most likely involved in the second step of catalysis [21]. Another artificial mutation identified is the replacement of the Asp residues with Asn or Glu in the central cavity of SQS, which completely eliminates its enzymatic activity, suggesting that the presence of these two Asp-rich motifs is fundamental for enzymatic activity [21]. Another key mutation point that has been identified across multiple species is lysine in codon 45. The K45R mutation has been related to higher cholesterol levels, making lysine 45 a potential exonic splicing enhancer site as it modulates cholesterol levels, possibly by altering intracellular cholesterol biosynthesis [29]. Mutagenic analysis of SQS through the deletion of amino acids in the N- and C-termini has revealed that the N-terminus has no role in catalysis; however, the catalytic domain of SQS is between residues 33 and 370 [6].

### 2.6. SQS Regulation

SQS enzymatic activity, protein levels, and mRNA expression are highly downregulated (90% suppression) by the end product of the mevalonate pathway, cholesterol, since it inhibits 98% of HMG-CoA reductase activity [6,30]. However, residual activity (2%) of this reductase is observed, even at high cholesterol levels, since some of the FPP products are necessary for proper cell growth [31]. To ensure the proper usage of FPP in this case, not continuing the mevalonate pathway, SQS is downregulated when cholesterol levels are high [31]. Conversely, low cholesterol levels have shown, alongside an increase in HMG-CoA activity, an 8-fold increase in SQS activity through the activation of the SQS promoter via the sterol regulatory element binding protein (SREBP) family, which act as transcription factors to maintain sterols homeostasis [6]. This group of transcription factors are bounded to the endoplasmic reticulum membrane when inactive [6]. However, when low sterol levels are detected, these are cleaved from the membrane and are hence activated [6]. The N-terminal domain of the protein enters the nucleus, where it binds the regulatory elements in the promoter region of genes coding for enzymes in the mevalonate pathway. Similarly to other genes associated with sterol biosynthesis, transcription of the FDFT1 gene is enhanced by maximal promoter activation through the accessory DNA-binding factors Sp1, NF-Y/CBF, and CREB as a complement to SREBPs [22]. Mutagenesis studies have identified three binding sites within 200 base pairs of the transcription initiation site (+1 site) in the hSQS gene [31]. These three binding sites have been associated with three SREBP family members: SREBp-1a, SREBP-2, and SREBP-1c/ADD-1. The first two have been shown to activate SQS transcription fully in experiments with transgenic mouse livers with overexpressed forms of SREBP; however, the latter one, SREBP-1c/ADD-1, did not produce a response [32,33,34]. Therefore, it has been concluded that SQS and the mevalonate pathway (cholesterol biosynthesis pathway) are regulated by sterols, specifically by the end product cholesterol, hence forming a negative feedback loop. As well as by cholesterol levels, SQS activity can be regulated by administering lipopolysaccharide (LPS), mimicking infection by gram-negative bacteria, which results in pneumonia, bloodstream infections, etc. [35] Similarly, SQS can also be regulated by the proinflammatory cytokines, tumor necrosis factor-α (TNF-α) and interleukin-1β (IL-1β) [36]. The effects of LPS and cytokines on cholesterol metabolism are different when looking at rodents and primates. In rodents, LPS, TNF-α, and IL-1β increase cholesterol levels, while, in primates, LPS and TNF-α have the opposite effect [37,38]. Experimental data have shown that the knockdown of the one of the two SQS genes in mice, named (ERG9) and (YHR190W), is lethal to the organism since squalene is essential for proper development and functioning of the central nervous system [39]. Additionally, SQS can be modulated at the protein level to regulate squalene synthesis and cholesterol synthesis [40]. Nevertheless, it has been predicted that SQS is not post-translationally modified [41]. Lastly, the reactant of SQS, FPP, has been shown to inhibit the enzyme at concentrations of 100 μM or higher [41]. It has been suggested that FPP acts as an inhibitor at high concentrations since it will start competing with PSPP for binding at the second active site located at the hydrophobic top end of the enzyme [42]. However, the intermediate between the first and second half-reactions, PSPP, does not inhibit SQS at any concentration [42]. This selective inhibition of SQS supports the structural model in which the catalytic sites are separate [43].

### 2.7. SQS Relevance in Health and Diseases

Despite SQS’s primary role in catalyzing the first reaction of the branch of the isoprenoid metabolic pathway, which is specifically committed to sterol biosynthesis, several studies have depicted the benefits in neoplastic pathologies by therapeutic intervention. Therefore, SQS has been identified as a potential therapeutic target for new treatments, including pharmacological intervention on this enzyme for prospective antineoplastic strategies. Some of the anticancer therapeutic strategies by SQS targeting have been identified, including non-small cell lung cancer [43,44], colon cancer [45], and prostate cancer [46,47]. In addition to the anticancer therapeutic strategies, interference with SQS and the isoprenoid pathway represent another interesting pharmacological intervention for other types of diseases, including Chagas disease [48], hepatitis C virus [49], and high cholesterol [50,51,52].

#### 2.7.1. Anticancer Therapeutic Strategies

##### Non-Small Cell Lung Cancer

Lung cancer accounts for 1 in 5 cancer deaths, making it the leading cause of cancer death in the U.S. [53]. Specifically, non-small cell lung cancer (including adenocarcinoma, large cell carcinoma, and squamous cell carcinoma) is more frequent (80% of lung cancer cases) than the other type, small lung carcinoma. [54] This disease is characterized by the formation of malignant cells in the tissues of the lung. Like other types of lung cancer, its major risk factor is smoking [55]. A biopsy can identify it, but common signs of non-small lung cancer are shortness of breath and persistent cough [56]. Previous studies have identified that enzymes, including SQS, participating in the cholesterol biosynthesis pathway are upregulated in lung cancer cells, and targeting them will inhibit migration and invasion [45]. Additionally, it has been reported that cancer cells are dependent on lipid metabolism (including the mevalonate pathway) to maintain their requirements of energy metabolism [44]. This information strongly suggests that these enzymes, including SQS, play a key role in non-small cell lung cancer progression. Particularly, SQS controls lipid rafts’ composition and promotes lung cancer metastasis by causing raft clustering in the cell membrane. Lipid rafts and high-cholesterol density plasma membranes modulate the assembly of signaling molecules and membrane fluidity. Therefore, SQS targeting has shown to inhibit lung cancer invasion/migration and metastasis in in vitro and in vivo studies [44]. Ultimately, targeting SQS may have considerable potential as a novel therapeutic strategy to treat lung cancer as an anticancer therapeutic strategy.

##### Colorectal Cancer

Colorectal cancer is a type of cancer that develops in the final part of the large intestine, the colon [56]. It is ranked second in mortality across the world [57]. The initial diagnosis is the appearance of polyps, benign clumps of cells, inside of the colon that then may develop into colon cancers [57]. Increased cholesterol levels have been correlated with the appearance of polyps. As we know, cancer cells rely on cholesterol to accommodate the increased demands in growth and nutrition during rapid cell division [45]. SQS is an upstream enzyme of SQLE in the sterol biosynthesis pathway and, ultimately, the formation of potential cancerous tumors [46]. FDFT1 induces the accumulation of oxygen-reactive species, causing the upregulation of SQS in stages I-III colon adenocarcinoma (COAD) [58]. High expression of FDFT1 was associated with patients at high risk of poor outcomes, establishing SQS and SQLE as accelerators for colon cancer cell proliferation and promoting tumor growth [59]. Conversely, lack of FDFT1 expression resulted in lower reactive oxygen species, inhibiting colon cancer cell proliferation due to the blockage of the pathway [59]. Therefore, the upregulation of SQS has emerged as a significant target for identifying a poor prognosis in stages I-III COAD, and its gene, FDFT1, is widely regarded as an oncogene. Ultimately, this can be transduced to the potential of targeting SQS as an anticancer therapeutic agent, since tumor cells are highly reliant on cholesterol and high cholesterol levels are correlated with the appearance of polyps.

##### Prostate Cancer

Prostate cancer is formed in the tissues of the prostate, which is a gland that forms part of the male reproductive system below the bladder [59]. In the U.S., 1 in 8 men are diagnosed with this type of cancer during their lifetime [60]. A recent study has shown how FDFT1 mRNA expression knockdown led to a significant decrease in prostate cancer cell proliferation [3,48]. In addition, individuals who have prostate cancer have shown a significantly higher level of FDFT1 mRNA expression than non-cancerous specimens have [48]. Therefore, the FDFT1 gene and its encoded enzyme, SQS, might become an important target in developing a pharmaceutical treatment for prostate cancer as an antineoplastic strategy. Further studies have also shown the ability to detect prostate cancer based on the levels of SQS observed in an MRI [47]. This second discovery opens the possibility for an accurate, standardized scoring system like more investigated cancers, such as breast and thyroid, have.

#### 2.7.2. Interference Therapeutic Strategies

##### Chagas Disease

The parasite *Trypanosoma cruzi* causes Chagas disease, which affects 8 million individuals in the world, and 300,000 in the U.S. [61]. Unlike Homo sapiens, Triatominae, also known as kissing bugs, use a variety of sterols, rather than cholesterol, as part of their cell membranes [49]. As known, SQS is part of the isoprenoid biosynthetic pathway, leading to sterol biosynthesis. For that reason, Chagas disease could be treated through the blockage of the sterol biosynthesis pathway through SQS inhibition. Several drugs blocking the SQS active site have been developed, such as SQ109 (Table 1, entry #7), which would allow the blockage of the sterol biosynthesis pathway [49].

##### Hepatitis C

Hepatitis C is a liver infection caused by the hepatitis C virus and spread through contact with blood from a patient who has previously contracted the infection. Most patients experience this illness for only a short time. Still, it becomes a long-term illness for more than half of infected individuals, sometimes even a chronic infection. Hepatitis C virus infection can cause threatening health conditions, including cirrhosis, severe liver scarring, and hepatocellular carcinoma and liver cancer [62]. SQS knockdown, through FDFT1 turn-off, led to a significant reduction of the hepatitis C virus, confirming the enzyme as an antiviral target [50]. Similarly to Chagas disease, the blockage of the sterol biosynthesis pathway was attempted by developing the YM-53601 drug as a potential SQS inhibitor [50].

##### High Cholesterol and Cardiovascular Diseases

Cardiovascular diseases (CVDs) are the leading cause of death for men, women, and people of most racial and ethnic groups in the U.S. [63]. Roughly 97 million U.S adults have cholesterol levels over 200 mg/dL, when the ideal levels are below 120 [52]. Elevated cholesterol levels have been identified as a risk factor for CVD due to increased fatty deposits in the walls of blood vessels, interrupting blood flow [64]. Cholesterol is not essential for most cells since it can be synthesized from acetyl-CoA via the mevalonate pathway. Increased cholesterol levels are treated by inhibiting 3-hydroxy-3-methylglutaryl coenzyme A (HMG-CoA) using statins, therefore blocking mevalonate production and the subsequent cholesterol biosynthesis pathway. Statins’ outstanding results in reducing between 30 and 58% of serum cholesterol [65] have made them the principal high cholesterol therapy. Nevertheless, blocking the cholesterol biosynthesis pathway at such an early step causes a decrease in mevalonate, FPP, and squalene. The production of these isoprenoid intermediates is required for proper lipid attachments for signaling molecules belonging to the Ras and GTPases family, the inhibition of which could result in some of the non-cholesterol-related effects of statins affecting migration, proliferation, and cytoskeletal structure. [65,66,67] Additionally, these intermediates are also key for the regulation of mitochondrial respiration (ubiquinone, coenzyme Q10), glycosylation (dolichol), protein synthase (isopentenyl adenine), and protein prenylation (geranylgeranyl diphosphate) [65]. Therefore, alternative therapies have been looked at to simulate statin therapy’s activity on cholesterol levels but with decreased side effects, including the decrease in the levels of non-sterol isoprene metabolites. In vitro studies have suggested that myotoxicity, a frequent side effect of statin therapy, is ceased by adding isoprenoid intermediates, such as farnesol, in the later steps of the pathway, which are then converted into FPP [68]. Additionally, FPP, a substrate of SQS, is the last water-soluble intermediate in this pathway, and its routes of metabolism are somewhat established [69]. Overall, myotoxicity is a current health concern. While statins are still used globally by 200 million patients, efforts to develop therapeutic drugs that can decrease cholesterol biosynthesis without affecting the isoprenoid metabolism are being made. 

### 2.8. SQS Small-Molecule Inhibitors

SQS has shown great potential as a therapeutic target for cancers. On theoretical grounds, it is considered the optimal point of the pathway for pharmaceutical intervention due to its substrate being water soluble with a decreased buildup of lipophilic and potentially toxic intermediates compared with other points in the pathway [7]. Therefore, researchers have tried to develop SQS inhibitors through various modes of action that could be used in cancer, as summarized in Table 1 and Table 2.

**Table 1:** SQS small-molecule inhibitors with crystal structure. pKi is the logarithm with base 10 of 1/ Ki (inhibitory constant, inhibitor concentration required to decrease the maximum reaction rate by half in mol/L) value. pIC_50_ is the logarithm with base 10 of 1/IC_50_ (half-maximal inhibitory concentration in mol/L).

Compound	X-ray Diffraction (Å)	Potency	Potency Indicator	PDB Number/ PubChem CID
Zaragozic Acid A [70,71,72,73]	1.89	10.1	pKi in rat liver cells	3VJC
Compound 21 [74]		11.4	pIC_50_ in rat microsomal SQS	10591006
Compound 15 a [75]	1.80	8.9	pIC_50_ in Hep-G2 cells	3V66
NB 598 [76]		7.7	pIC_50_ in rat liver cells	6443223
Compound 7 [77]	2.00	5.19	pIC_50_ in rat microsomal SQS	3ASX
N-[(3R,5S)-7-Chloro-5-(2,3-dimethoxyphenyl)-1-neopentyl-2-oxo-1,2,3,5-tetrahydro-4,1-benzoxazepine-3-acetyl]-L-aspartic acid [78]	2.00			3Q2Z
SQ-109 [78]	2.90	6.1	pKi in *Staphylococcus aureus*	3WSA
E5700 [79]	2.32	8.8	pIC_50_ in rat liver cells	3WCC
ER-119884 [80]	2.75	1.1	pIC_50_ in rat liver cells	3WCE
BPH1344 [49]	2.80	6.59	pIC_50_ in purified hSQS	3WCG
BPH1218 [49]	2.22	7.28	pIC_50_ in purified hSQS	3WCF
BPH1237 [49]	2.50	7.05	pIC_50_ in purified hSQS	3WCH
BPH1325 [49]	2.30	6.57	pIC_50_ in purified hSQS	3WCI
WC-9 [80]	2.75	7.06	pIC_50_ in T. cruzi	3WCD
BPH652 [81]	2.00	9.70	pKi in rat microsomal SQS	3LEE

## 3. SQS in Ferroptosis

### 3.1. Overview of Ferroptosis

Cells can die in two separate ways: accidental cell death (ACD) or regulated cell death (RCD) [129]. RCD is a highly controlled and regulated process that includes signaling cascades, and it is required for tissue homeostasis so that organisms can regulate cell proliferation and cell death, and inhibit tumor growth [130,131]. RCD is common in diseases ranging from neurodegenerative diseases to cancers. The earliest discovered form of RCD, in 1972 by John Keer et al., is apoptosis [132]. However, a recurring problem with apoptosis is cancer cells’ resistance to this type of cell death when using anticancer drugs [133,134]. The increasing amount of research in medicinal chemistry has led to a detailed list of RCD mechanisms, especially in developing therapeutics for cancers and other diseases [135]. Previously, cell death mechanisms in mammals were classified as apoptosis, necroptosis, or autophagy-dependent; however, new forms of RCD have been discovered, including pyroptosis [136] and ferroptosis [56]. Ferroptosis was discovered when a group of scientists were developing therapeutic drugs targeting a mutated proto-oncogene involved in cancer, RAS [50,56]. These researchers were able to find two compounds toxic to cancer cells expressing mutated RAS, RSL3, and erastin [50,56]. The type of cell death followed by the treatment with these two drugs did not fit into any of the already discovered RCD mechanisms [50,56]. Since this cell death mechanism was shown to be ineffective in the presence of deferoxamine, an iron chelator, ferroptosis was then defined as an iron-dependent process that originates membrane damage through lipid peroxidation [47,50,56]. Ferroptosis has been linked with multiple diseases, since inducing this type of RCD seems to be a potential therapy for Alzheimer’s disease, Parkinson’s disease, Huntington’s disease, lung cancer, prostate cancer, breast cancer, melanoma, hepatocellular carcinoma, pancreatic cancer, and skin cancer. Therefore, the therapeutic potential of ferroptosis in this field is immense, and its relation to other diseases is also emerging. Back in the 1950s, Harry Eagle was able to demonstrate how cell death was achieved by cysteine depletion and the consequent reduction of glutathione. In contrast, cysteine synthesis did not reduce glutathione but restored its levels, causing cell death cessation [137,138]. A few years after, a type of vitamin E, alpha-tocopherol, was identified by Shiro Bannai et al. as an antioxidant capable of reversing cell death, regardless of the glutathione levels and no cystine present [139]. Finally, in 2012, “ferroptosis” was distinguished from other RCD forms and was defined as glutamate-induced cell death [140]. Ferroptosis was characterized as sensitive to iron and lipid peroxidation due to its inefficacy in the presence of an iron chelator and an antioxidant, respectively. In general, ferroptosis results in morphological changes, including a reduced mitochondrial volume, increased density of the lipid bilayer membrane, disrupted outer mitochondrial membrane, dwindled mitochondrial cristae, swelled cells, and ruptured plasma membrane [141,142]. Due to the increased metabolic demand for iron to support cancer cell growth, ferroptosis can be used for therapeutic purposes. Therapeutic strategies can disrupt this metal homeostasis and therefore trigger ferroptosis, resulting in an inhibition of cell proliferation, cell death, and tumor growth. It is well known that tumors have an increased metabolic demand for iron to support their growth, and this could be turned to therapeutic purposes by deregulating the metal homeostasis and triggering this type of programmed cell death. Biochemically, ferroptosis leads to glutathione depletion, a subsequent decrease in GPX4 activity, mevalonate-pathway-derived coenzyme Q10 (CoQ_10_) depletion, and consequent SQS activation [143,144,145]. Additionally, the mevalonate pathway final product, cholesterol, has been closely related to this programmed cell death type. Abundant cholesterol metabolites’ availability has shown to increase the capacity of tumor and metastatic cells by upregulating cellular uptake and lipid biosynthesis [146]. This relation is not surprising as cholesterol composes part of cell membranes and is extremely susceptible to oxidation, especially by hydroxyl radicals [146]. These findings foreshadow the role that SQS can potentially play in the regulation of ferroptosis and, therefore, in the therapeutic field across many diseases.

### 3.2. Molecular Mechanisms of Ferroptosis

Ferroptosis is driven by an imbalance in the levels of oxidants and antioxidants that results in lipid peroxidation, a process by which free radicals or reactive oxygen species (ROS) attack lipids containing double-bounded carbons (C=C), especially polyunsaturated fatty acids (PUFAs) present in the cell membrane [143,147] Cells often resolve lipid damage through phospholipid peroxidase glutathione peroxidase 4 (GPX4) as an antioxidant-reducing toxic hydroperoxide [148]. Ferroptosis is characterized by the production and accumulation of lipid peroxides and the failure of internal mechanisms to eliminate them [149]. One potential explanation for this phenomenon involves the suppression of GPX4, thereby triggering a process known as ferroptosis cell death [147]. The resulting accumulation of lipid peroxides reaches lethal levels, which causes damage to the phospholipids conforming the lipid bilayer of the cell membrane, rupturing the cell and triggering ferroptosis cell death [150]. Three main regulatory levels of ferroptosis have been identified: (1) system Xc/reduced glutathione/glutathione peroxidase 4, (2) nicotinamide adenine dinucleotide phosphate/ferroptosis suppressor protein 1/coenzyme Q10 (CoQ10), and (3) guanosine triphosphate (GTP) cyclohydrolase 1/tetrahydrobiopterin/dihydrofolate reductase [149,151]. Regulatory systems (1) and (2) have been shown to be downregulated when SQS is active, as shown by the usage of class III ferroptosis inducers (FINs) [151,152]. For regulatory level (1), once SQS is activated through the binding of FIN56, GPX4 is depleted and inactivated, resulting in the rapid accumulation of ROS. Nevertheless, these lipid ROS can be repressed with iron chelators and lipophilic radical traps [152]. For regulatory level (2), the available FPP suffers competition between the different paths that it can take, including the formation of CoQ10 or the reaction on SQS to form squalene, based on the schematics of the mevalonate pathway (Scheme 1) [153].

### 3.3. Regulation of Ferroptosis through SQS

Squalene is an oleophilic metabolite that accumulates in lipid droplets or cell membranes, especially in anaplastic large cell lymphoma (ALCL) cell lines. Hence, ferroptotic cell death and lipid peroxidation can potentially be regulated by SQS, similarly to GPX4 [44]. Cell lines not expressing SQLE, commonly ALCL, experience an accumulation of squalene due to the non-use of it in the cholesterol biosynthesis pathway (mevalonate pathway). SQLE is directly downstream from SQS in the mevalonate pathway. Through CRISP-9 experiments, SQS knockdown results in a decrease of squalene storage back to non-ALCL cell levels. Other experiments showed decreased tumor volume and size when knocking down FDFT1 due to removing squalene accumulation. Overall, these results suggest that SQS promotes optimal growth in ALCL cells through squalene accumulation [44]. Silencing FDTF1 and blocking squalene accumulation resulted in ferroptosis in ALCL cells when GPX4 was inhibited [44]. Supplementing the cells with extracellular squalene did not balance out FDFT1 knockdown, indicating that squalene must accumulate in the correct compartments, ER membranes, to protect against ferroptotic cell death [44]. These findings were further supported using an antioxidant (ferrostatin-1), which showed a decreased sensitivity to GPX4 inhibitors in FDFT1 knockdown ALCL cells [44]. Similarly, expression of SQLE in ALCL cells removing the squalene accumulation resulted in a decrease in tumor growth [44]. Removal of the squalene accumulation via FDFT1 knockdown, SQLE expression, or SQS inhibition has been associated with an overexpression of lipid ROS, a feature of ferroptosis [44]. FIN56, a small-molecule inducer of ferroptosis, has been tested on the human fibrosarcoma HT1080 cell line (these cells are from human epithelial tissue obtained from a fibrosarcoma patient) [154], and through short hairpin RNAs (shRNAs) against FDFT1. The data suggest that FIN56 activates SQS. Additionally, inhibiting SQS with known inhibitors (YM-53601 and zaragozic acid A) has shown a suppression in FIN56 lethality. The binding of hSQS protein to an affinity probe vanished by pre-incubation of purified SQS with FIN56, suggests a binding interaction between SQS and FIN56 [154]. It is also known that SQS’s substrate, FPP, participates in other processes besides cholesterol biosynthesis, such as synthesizing sterols, CoQ10, dolichol, ubiquinone, and heme A. Supplementation of FPP, SQS inhibition, and SQLE inhibition separately suppressed FIN56 lethality in HT1080 cells [154]. These results suggest the idea of certain metabolites derived from FPP regulating the cytotoxicity of FIN56. CoQ10 was the only one to suppress cell death via FIN56-induced ferroptosis among the four known metabolites. Thus, CoQ10 is thought to regulate FIN56 activity. However, CoQ10 supplementation has not been effective due to its extreme hydrophobicity and SQS being in a hydrophilic environment [50]. The combination of these results suggests that squalene can protect against chemical modifications when PUFAs membranes are damaged via oxidation. Loss of squalene accumulation in the ER membrane has also shown the depletion of PUFAs membranes. Although GPX4 is considered protective against lipid peroxidation, no change in GPX4 protein levels has been observed with FDFT1 knockdown. Researchers have concluded that squalene is an upstream metabolite in the cholesterol biosynthesis pathway that protects against peroxidation, whereas its absence promotes ferroptosis [44,47,50]. Additionally, CoQ10 is thought to regulate FIN56 lethality, but its supplementation as an effective technique to suppress ferroptosis is held back due to its extreme hydrophobicity. In other words, squalene is considered to possess anti-ferroptosis properties in certain cancer cells [44].

#### Conclusions and Perspectives

Squalene synthase has been launched into the research field as a potential therapeutic target because its product, squalene, protects cellular membranes from oxidative damage and participates in the mevalonate pathway. Its potential protective function against ferroptosis and lipid peroxidation is cognate to the accumulation of squalene in the endoplasmic reticulum membrane, which acts as an antioxidant and a lipophilic radical trap. Accordingly, SQS has been identified as a regulator of not only ferroptosis cell death pathways but also other diseases, ranging from viruses to cancers. In the past 20 years, an increasing amount of research has been conducted on SQS, which has been discovered in an extensive number of inhibitors and modulators. Even though its biochemical structure, reaction mechanism, significance in diseases, and relation to ferroptosis have been discovered, much remains to be learnt about the regulation and therapeutic usage of SQS. Increasing the knowledge on how SQS inhibition in humans could be achieved as an alternative assignment for other treatments such as chemotherapy, radiotherapy, and statins. Due to the challenges experienced during past clinical trials, there is a limitation in the current inhibitors. The continued development of new SQS inhibitors with improved drug-like and pharmacokinetic properties and their ultimate clinical trials may facilitate the pharmaceutical development of compounds targeting this enzyme. Therefore, the research field hopes that a better knowledge of SQS’s regulation and therapeutic usage can promote clinical development in treating cancers and other conditions.

## Figures and Tables

**Table 2:** Reported novel inhibitors of SQS. pKi is the logarithm with base 10 of 1/Ki (inhibitory constant, inhibitor concentration required to decrease the maximum reaction rate by half in mol/L) value. pIC_50_ is the logarithm with base 10 of 1/IC_50_ (half-maximal inhibitory concentration in mol/L).

Compound	Potency	Potency Indicator	PubChem CID
Squalestatin 2/Zaragozic Acid B [74]	10.5	pKi in Hep-G2 cells	9940176
Squalestatin 3/Zaragozic Acid C [74]	10.4	pKi in Hep-G2 cells	11814656
Compound 5d [82]	10.4	pIC_50_ in rat microsomal SQS	44352892
Compound 19 [75]	10.3	pIC_50_ in rat microsomal SQS	10662370
L735021 [83]	9.9	pIC_50_ in rat microsomal SQS	9853075
Compound 505374 [83]	9.52	pIC_50_ in Hep-G2 cells	
Compound 33b [76]	9.29	pIC_50_ in rat liver cells	
Compound 6 [84]	9.0	pIC_50_ in S. aureus	56947056
SQ34919 [85]	9.0	pIC_50_ in rat microsomal SQS	10382597
RPR 101821 [86]	9	pIC_50_ in rat microsomal SQS	
Compound 22a [78]	8.82	pIC_50_ in rat liver cells	
Compound 1e [87]	8.7	pKi in rat microsomal SQS	44370557
J104123 [88]	8.6	pIC_50_ in dogs	9848748
Compound 2d [86]	8.6	pIC_50_ in rat microsomal SQS	10405846
Compound 23 [89]	8.5	pIC_50_ in rat microsomal SQS	19077552
ER-28448 [80]	8.44	pIC_50_ in rat liver cells	11540104
Compound 7 [90]	8.4	pIC_50_ in rat liver cells	10358175
Compound 11 [91]	8.30	pIC_50_ in *T. cruzi*	
Compound 8 [92]	8.1	pIC_50_ in rat liver cells	98110129
Compound 16a [93]	8.0	pIC_50_ in rat microsomal SQS	44373472
Compound 5j [94]	7.9	pIC_50_ in rat liver cells	10699948
Compound 4ª [95]	7.89	pIC_50_ in rat microsomal SQS	
Compound 4 [96]	7.8	pIC_50_ in rat liver cells	56947060
Compound 1 [97]	7.7	pIC_50_ in rat microsomal SQS	19956992
CP-424677 [9]	7.49	pIC_50_ in rat microsomal SQS	
A-87049 [96]	7.43	pIC_50_ in rat liver cells	
Compound 6 [98]	7.4	pKi in rat microsomal SQS	44370654
CP-320473 [9]	7.25	pIC_50_ in rat microsomal SQS	
Compound 11 [99]	7.22	pKi in human liver cells	
Compound 4q [77]	7.2	pIC_50_ in rat microsomal SQS	10411224
YM 175 [100]	7.19	pIC_50_ in rat liver cells	119188
YM53579 [101]	7.10	pIC_50_ in rat microsomal SQS	11372147
Chlorogenic acid [102]	7	pIC_50_ in pig liver cells	1794427
BPQ-OH [103]	6.96	pIC_50_ in rat liver cells	9817140
Compound 15 [104]	6.9	pIC_50_ in rat microsomal SQS	44370656
CP294838 [105]	6.9	pIC_50_ in rat liver cells	9889227
TAK475 (Lapaquistat) [106]	6.82	pIC_50_ in Hep-G2 cells	9960389
Compound 23 [107]	6.77	pIC_50_ in rat liver cells	
P-3622 [18]	6.70	pIC_50_ in Hep-G2 cells	
EB 1053 [101]	6.68	pIC_50_ in rat liver cells	130821
Compound 14 [108]	6.6	pIC_50_ in rat liver cells	44382842
L731128 [73]	6.6	pIC_50_ in rat liver cells	9931928
PHPBP [101]	6.51	pIC_50_ in rat liver cells	
Compound 15 [108]	6.5	pKi in purified hSQS	44185382
MPEX098 [109]	6.39	pIC_50_ in purified hSQS	
BPH830 [109]	6.3	pKi in purified hSQS	44182294
Compound 4g [110]	6.2	pIC_50_ in *L. donovani*	44584870
Schizostatin [111]	6.08	pIC_50_ in rat liver cells	9862523
Compound 9 [112]	6.0	pIC_50_ in rat microsomal SQS	25147760
DF-461 [113]	5.96	pIC_50_ in rat liver cells	57777744
CJ-13,982 [114]	5.96	pIC_50_ in rat liver cells	10428617
2*R*,3*S* diphosphate enantiomer [21]	5.93	pIC_50_ in yeast	
Compound 17 [115]	5.7	pIC_50_ in rat microsomal SQS	10475079
CJ-981 [115]	5.55	pIC_50_ in rat liver cells	
EP2302 [116]	5.52	pIC_50_ in Hep-G2 cells	
Compound 19 [117]	5.48	pIC_50_ in human liver	
Compound 20 [118]	5.4	pIC_50_ in rat microsomal SQS	46866079
BMS-188494 [69]	5.39	pIC_50_ in rat microsomal SQS	154098
BMS-187745 [69]	5.16	pIC_50_ in rat microsomal SQS	153978
EP2306 [117]	4.88	pIC_50_ in Hep-G2 cells	
Carnosol [119]	4.75	pIC_50_ in rat liver cells	442009
CP-458003 [9]	4.52	pIC_50_ in rat microsomal SQS	
ER-27856 [120]	4.41	pIC_50_ in rat liver cells	9896881
CLSPHSMFC [121]	4.19	pIC_50_ in Hep-G2 cells	
SMFC [122]	4.12	pIC_50_ in Hep-G2 cells	5272743
YM-53601 [122]	4.10	pIC_50_ in Hep-G2 cells	9907532
CKTE [122]	4.06	pIC_50_ in Hep-G2 cells	
WHQW [122]	4.05	pIC_50_ in Hep-G2 cells	
CJ-15,183 [115]	4.01	pIC_50_ in human liver cells	9894585
Compound 5 [123]	8.14–8.24	pIC_50_ in Hep-G2 cells	
RPR 107393 [124]	6.05–6.22	pIC_50_ in rat liver cells	10314587
Compound 12 [125]	6.6–6.8	pIC_50_ in rat liver cells	56947012
J104118 [126]	9.1–9.3	pIC_50_ in rat microsomal SQS	10460101
(3-{[1-(prop-2-en-1-yl)-9H-carbazol-2-yl]oxy}propyl)(propan-2-yl)amine [127]	7.2–7.5	pIC_50_ in Hep-G2 cells	9949081
Compound 1 [128]	5.2–7.0	pIC_50_ in rat liver cells	10409462

## Abbreviations

SQS	Squalene synthase
ROS	Reactive oxygen species
FPP	Farnesyl pyrophosphate
SQLE	Squalene epoxidase
FPS	Farnesyl-pyrophosphate synthase
Asp-RR	Aspartate-rich sequence motif regions
PSQPP	Presqualene pyrophosphate
NADPH	Nicotinamide adenine dinucleotide phosphate
PPi	inorganic phosphate
FDFT1	Farnesyl-diphosphate farnesyltransferase 1
hSQS	Human squalene synthase
PDB	Protein Data Bank
ER	Endoplasmatic reticulum
SQSD	Squalene synthase deficiency
(TNF-)	Tumor necrosis factor-*α*
(IL-1*β*)	Interleukin-1*β*
COAD	Colon adenocarcinoma
CVD	Cardiovascular disease
HMG-CoA	Methylglutaryl coenzyme A
CID	Compound identifier
ACD	Accidental cell death
RCD	Regulated cell death
ROS	Reactive oxygen species
PUFAs	Polyunsaturated fatty acids
GPX4	Phospholipid peroxidase glutathione peroxidase 4
CoQ10	Coenzyme Q10
GTP	Guanosine triphosphate
FINs	Ferroptosis inducers
ALCL	Anaplastic large cell lymphomas
shRNAs	Short hairpin RNAs
